# The use of high-flow nasal oxygen vs. standard oxygen therapy in hematological malignancy patients with acute respiratory failure in hematology wards

**DOI:** 10.3906/sag-2007-228

**Published:** 2021-08-30

**Authors:** Nilgün APLTEKİNOĞLU MENDİL, Şahin TEMEL, Recep Civan YÜKSEL, Kürşat GÜNDOĞAN, Bülent ESER, Leylagül KAYNAR, Murat SUNGUR

**Affiliations:** 1 Department of Critical Care Medicine, Erciyes University Medical School, Kayseri Turkey; 2 Division of Hematology, Department of Internal Medicine, Erciyes University Medical School, Kayseri Turkey

**Keywords:** Acute respiratory failure, high flow nasal oxygen, hematological malignancy

## Abstract

**Background/aim:**

High flow nasal cannula (HFNC) was mostly used in intensive care units (ICUs) with few studies in other departments. We hypothesized that HFNC applied at wards is beneficial for acute respiratory failure in hematological malignancy patients.

**Materials and methods:**

The study is a single center, randomized controlled study. Inclusion criteria were hypoxemic respiratory failure and hematological malignancy. Patients were randomized to either venturi mask/nasal cannula oxygen treatment or HFNC.

**Results:**

One hundred patients were included in the study. Median age was 58.5 (18–86) years and APACHE II score was 17 (5–29). HFNC group was 51 patients and the oxygen treatment group 49 patients. P/F ratios were similar between the groups throughout the study period. Endotracheal intubation was required in 10 (20.0%) patients in oxygen group and 17 (33.0%) patients in HFNC group (p = 0.14). A total of 17 (35.0%) patients in oxygen group and 17 (33.0%) patients in HFNC group received noninvasive mechanical ventilation (p = 0.97). Median VAS comfort scores at the 2nd and 24th hours were not different between groups. The 28-day mortality rate was 36.7% (18 deaths) in the standard group and 45.0% (23 deaths) in the HFNC group (p = 0.39).

**Conclusion:**

HFNC applied in wards is not superior to standard oxygen treatment for acute respiratory failure in hematological malignancy patients.

## 1. Introduction

New and effective treatment techniques for hematological patients introduced during the last decade improved survival and disease-free periods [1,2]. Increasing number of hematological malignancy patients require intensive care unit (ICU) admissions with improved survival and immunosuppression related problems [3]. 

Acute respiratory failure is the leading cause for ICU admission for immunocompromised and especially for hematological patients [4]. Endotracheal intubation and invasive mechanical ventilation requirement are associated with very high mortality rates up to 70% in these subgroup of patients [5]. This prompted to seek alternative techniques of respiratory support to determine if it is possible to avoid from invasive mechanical ventilation [5–7]. Respiratory support in early stages of hypoxemic acute respiratory failure in immunocompromised patients was debated before [5,8,9]. In the largest randomized controlled study reported in 2015, early noninvasive ventilation compared with oxygen therapy alone did not reduce mortality, ICU-acquired infections, duration of mechanical ventilation, or lengths of ICU or hospital stays in this subset of the patients [10]. However, authors declared that the study was underpowered. 

Oxygen treatment either with nasal cannula or face mask is the first step treatment in hypoxic respiratory failure to improve oxygenation. Flow rates are limited with these devices because these devices are unable to achieve appropriate level of heat and humidity with high flow rates. The fraction of oxygen given to the patient is also highly variable with these devices. High flow oxygen devices are available during the last decade that can deliver oxygen up to 60 L/min with active heated humidification [11]. The fraction of oxygen is also can be adjusted with an oxygen blender incorporated into the system. High flow oxygen provided with a nasal cannula flushes out carbon dioxide from anatomical dead space and decreases respiratory rate [12]. High flow nasal oxygen also provides greater comfort, tolerability, better respiratory pattern and oxygenation compared to other low flow system devices. High flow nasal oxygen was proved to be beneficial in various forms of acute respiratory failure patients [13–17] 

As application of high flow nasal oxygen in adult ICUs is relatively recent, the evidence supporting its use remains limited and is composed of predominantly observational studies [18]. There are also a limited number of studies in immunocompromised patients [19]. A recent randomized controlled study in immunocompromised patients showed that high flow oxygen therapy did not significantly decrease 28-day mortality and the need for endotracheal intubation when compared with standard oxygen therapy [20]. Most studies on high flow nasal oxygen in patients with hypoxemia have been conducted in an intensive care unit or high-dependency unit. There are discussions if high flow nasal oxygen for acute respiratory failure should be used only in intensive care units or not [21].

We hypothesized that HFNC performed in hematology wards can decrease need for invasive, noninvasive mechanical ventilation and need for ICU admission in acute respiratory failure of hematological malignancies. 

## 2. Patients and methods

The study is a single center, prospective, open label randomized controlled study comparing standard oxygen treatment vs. high flow nasal oxygen (HFNC) in early phase of acute hypoxemic respiratory failure of hematological malignancy patients. Patient recruitment was performed between November 2016 and September 2018. The study was conducted in a university hospital in Kayseri, Turkey. The study protocol was approved by Erciyes University ethics committee (2016/596). Written informed consent was obtained from all patients. 

### 2.1. Patients

The patients were recruited in hematology and bone marrow transplantation clinics of Erciyes University Hospital. Eligible patients were hematological malignancy patients regardless of time from diagnosis or bone marrow transplant patients with signs of respiratory distress or labored breathing. Inclusion criteria were (1) Patients above 18 years of age, (2) PaO_2_/FiO_2 _< 300 mmHg or oxygen saturation by pulse oximetry (SpO_2_) < 92% on room air, (3) PaCO_2_ ≤ 45 mmHg, (4) respiratory rate > 22 breaths/min or labored breathing with respiratory distress. 

Exclusion criteria were (1) Patients refused to enter into the study, (2) Pregnant or breast-feeding patients, (3) Need for noninvasive (NIMV) or invasive mechanical ventilation (IMV) at the time of randomization, (4) Need for intensive care unit (ICU) admission at the time of randomization, (5) PaCO_2_ > 45 mmHg, (6) Hemodynamic instability (mean arterial pressure less than 65 mmHg and/or need for vasopressors), (7) Cardiogenic pulmonary edema, (8) Patients unable to cooperate. 

### 2.2. Randomization 

Clinicians participating in the study were randomly given sealed and opaque envelopes containing treatment allocation as either high-flow nasal oxygen therapy or standard oxygen therapy. The envelope was opened, and the allotted treatment given after the patient gave its written informed consent.

### 2.3. Treatments

Treatments other than oxygen and HFNC treatment were made by clinical team caring for these patients according to their standard practice for both groups. Oxygen or HFNC treatments were initiated within 30 min after randomization. Respiratory treatments such as respiratory therapy and bronchodilators were also managed by the physicians caring for the patients. 

### 2.4. High-flow oxygen treatment

Initial flow rate was started as 30 L/min with FiO_2_ of 100% and flow rate increased up to 50 L/min as for patient’s tolerability. FiO_2_ is adjusted to keep SpO_2_ ≥ 94%. Minimal flow rate used is 30 L/min during the study period. HFNC is used as much as possible during the study period including mealtimes, procedures and transfers. HFNC was weaned per patient’s improvement in clinical signs. We have started give breaks if PaO_2_/FiO_2_ ≥ 350 and ability to keep SpO_2_ ≥ 94% with nasal cannula or face mask oxygen without any signs of respiratory distress.

### 2.5. Oxygen treatment

Oxygen was provided with nasal prongs or facial oxygen masks without reservoir bag. Initial oxygen flow was 6 L/min and adjusted to keep SpO_2_ ≥ 94 %. HFNC is never used in standard oxygen treatment patients. NIMV or IMV with endotracheal intubation is used in case of failure based the criteria described below. 

### 2.6. Treatment failure

Treatment failure was diagnosed in case of (1) metabolic or respiratory acidosis, (2) FiO_2_ ≥ 60% to keep SpO_2_ ≥ 92 %, (3) hemodynamic instability (vasopressor requirement to keep mean arterial pressure (MAP) ≥ 65 mmHg, severe tachycardia or bradycardia, (4) neurological disorientation. 

All failed patients were transferred to the medical ICU and either NIMV or IMV was applied per ICU team. 

### 2.7. Data collection

We have screened all patients in hematology ward and bone marrow transplant unit daily, at least ones during the study period. Hematology team was aware of the study and we were called by them in case of possible new patient inclusion at other times of the day. 

We have recorded baseline characteristics of the patients including vital signs, hematological diagnoses, activity status of the hematological disease, reason for respiratory insufficiency, APACHE II score, SOFA score, co-morbid conditions, baseline laboratory values, visual analog scale (VAS) for dyspnea, comfort, and sensation of thirst. VAS scores are collected at the 2nd and 24th hours after initiation of oxygen or HFNC therapy. Baseline arterial blood gas and vital signs were recorded. Arterial blood gas and vital signs were collected at least ones a day (worst values were recorded). SpO_2_ was monitored continuously. 

Mortality at 28 days, need for ICU transfer, endotracheal intubation, NIMV, hospital and ICU length of stay were all assessed at the end of the study. 

### 2.8. Outcomes

The primary goal of the study to show if HFNC can decrease need for endotracheal intubation (first 7 days) for each group. The secondary goals of the study to show if there is an improvement in mortality (28 days) and need for invasive and noninvasive ventilation as well as improvement of VAS score for, dyspnea, and sensation of thirst (first 7 days) for each group. 

### 2.9. Statistical analysis

All randomized patients were included into the final analysis. The patients were randomized to either standard medical treatment or HFNC treatment groups. All statistical analyses were calculated by using IBM Statistic SPSS v: 22.0 (Chicago, IL, USA). Data are expressed as the median (including the lower and upper quartiles). Comparisons between groups for continuous variables were performed using the Mann–Whitney U test. The χ^2^ test was used to determine significant differences in proportions among categorical variables. A p value of 0.05 and lower was considered statistically significant.

## 3. Results

 We screened 650 patients and we included 102 patients into the study. Two patients in HFNC group developed epistaxis immediately after initiation of HFNC excluded from the study and 100 patients completed the study. The median age was 58.5 (18–86) years and 66 patients were male 34 patients were female. The most common reason for exclusion was absence of respiratory insufficiency (436 patients). Of the patients who were enrolled, 53 patients randomly assigned to the HFNC group and 49 patients to the control group. (Figure). Baseline characteristics of the patients are shown in Table 1. There was a statistically significant difference in hemoglobin and APACHE-II levels between the two groups. All patients had a diagnosis of hematological malignancy. The most common hematological malignancies were acute myelomonocytic leukemia and lymphoma with 43 and 26 patients respectively. The malignancies were active in 55 patients, in remission in 12 patients, relapsing disease in 32 patients, and one patient had graft versus host disease. None of the patients had do not intubate orders. The most common reason for respiratory failure was pneumonia and extra pulmonary sepsis in 74 and 10 patients, respectively. 

**Table 1:** Patients demographic, clinical and laboratory characteristics.

	Totaln=100	Intervention group(High flow) n=51	Control group(Standard O2) n=49	P
Age, years (min-max)	58.5 (18-86)	58 (18-86)	59 (27-81)	0.586*
Gender, n (%)				0.780**
Male	66(66)	33(65)	33(67)	
Female	34(34)	18(35)	16(33)	
Hematologic disease, n (%)				0.186**
AML	43(43)	24(47)	19(39)	
Lymphoma	26(26)	13(25)	13(26)	
Multiple Myeloma	19(19)	6(12)	13(26)	
MDS	6(6)	5(10)	1(2)	
ALL	4(4)	1(2)	3(7)	
KLL	1(1)	1(2)	0(0)	
KML	1(1)	1(2)	0(0)	
Reason for acute respiratory failure, n (%)				
Pneumonia	74(74)	40(78)	34(70)	0.424**
Extra pulmonary Sepsis	10(10)	6(12)	4(8)	
Pleural effusion	7(7)	3(6)	4(8)	
Non-infections pulmonary disease	4(4)	0(0)	4(8)	
CHF	2(2)	1(2)	1(2)	
ARDS	1(1)	0(0)	1(2)	
Other	2(2)	1(2)	1(2)	
APACHE II score,median (min-max)	17 (5-29)	18 (5-25)	16 (7-29)	0.014*
SOFA score, median (min-max)	6 (0-10)	6 (2-10)	5 (0-10	0.112*
GCS, median (min-max)	15 (12-15)	15 (13-15)	15(12-15)	0.715*
CCI, median (min-max)	3 (1-10)	3 (2-9)	3 (1-10)	0.282*
PaO2/FiO2 ratio, median (min-max)	262 (190-295)	257 (209-295)	276 (190-295)	0.073*
pH, median (min-max)	7.48 (7.31-7.59)	7.48 (7.31-7.59)	7.49 (7.36-7.59)	0.178*
PO2, median (min-max)	55.1 (40-62)	53.4 (44-62)	58 (40-62)	0.062*
PCO2, median (min-max)	30 (17-41)	30 (17-41)	32.5 (19-41)	0.444*
HCO3, median (min-max)	23 (11.6-31.6)	23.3 (13.9-30.6)	23 (11.6-31.6)	0.646*
Lactate, mmol/L median (min-max)	1.43 (0.57-8.40)	1.50(0.6-4.8)	1.40(0.57-8.4)	0.497*
Hemoglobin concentration, median (min-max)	8.45 (4.5-13.6)	8.2 (4.5-11.3)	8.7 (6.3-13.6)	0.030*
White blood cell count/μL, median (min-max)	4985 (0.0-169300)	5580 (0.0-169300)	4060 (0.0-93390)	0.409*
Platelet count/μL median (min-max)	34000 (4000-385000)	33000(6000-290000)	35000 (4000-385000)	0.581*

AML: acute myelomonocytic leukemia, MDS: myelodysplastic syndrome, ALL: acute lymphocytic leukemia, CLL: Chronic lymphocytic leukemia, CML: chronic myelomonocytic leukemia, CCI: Charlson comorbidity index.*Comparisons between groups were performed using the Mann–Whitney U test. **Comparisons between groups were performed using the chi-square test.

**Figure:**
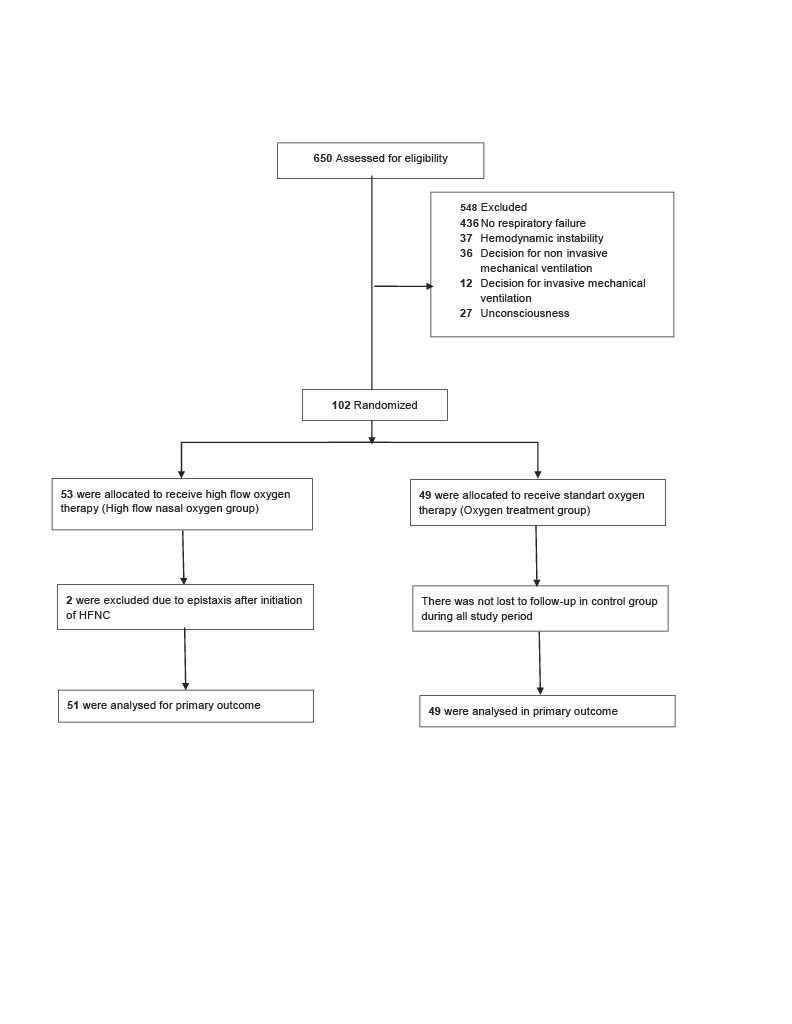
Patient flow chart. HFNC: high flow nasal oxygen.

At the time of randomization, median APACHE II score was 18 (5–25) and 16 (7–29) (p = 0.014) and SOFA score was 6 (2–10) and 5 (0–10) (p = 0.112) in HFNC and oxygen therapy groups respectively. Charlson comorbidity index was 0 in 45 patients and 1–2 in 40 patients, 15 patients had an index of 3 and above. 

At the time of randomization median PaO_2_/FiO_2_ was 257 mmHg (209–295) and 276 mmHg (190–295) in HFNC group and oxygen therapy group respectively. All patients in HFNC group received high flow oxygen immediately after the randomization. The median flow rate with HFNC was 40 (20–50) L/min and median FiO_2_ was 0.35 (0.28–0.60). All 51 patients with HFNC were able tolerate the treatment. Oxygen was given with using face mask and nasal cannula to the patients in control group and the median flow rate was 5 (1–15) L/min. 

### 3.1. Primary outcomes

Endotracheal intubation and invasive mechanical ventilation were required in 17 (33%) patients in HFNC group and 10 (20%) patients in oxygen group (p = 0.14). Need for noninvasive mechanical was observed in 17 (33%) and 17 (35%) patients in HFNC group and control groups respectively (p = 0.88). VAS for comfort, thirst, and dyspnea were not different between the groups (Table 2).

**Table 2:** Patient’s clinical outcomes.

Variables	High Flow Nasal Oxygen Treatment n=51	Standard Oxygen Theraphyn=49	P*
Need for intubation, n (%)	17 (33)	10 (20)	0.146
Need for non-invasive MV, n (%)	17 (35)	17 (33)	0.886
VAS, Dispne (min-max)			
2 hours	6 (1-9)	6 (0-9)	0.481
24 hours	4 (0-7)	4 (0-9)	0.984
VAS, Comfort (min-max)			
2 hours	6 (2-9)	6 (0-9)	0.182
24 hours	4 (0-10)	4 (0-9)	0.857
VAS, Thirst (min-max)			
2 hours	4 (0-10)	5 (0-9)	0.957
24 hours	3.5 (0-8)	4 (0-9)	0.307
Need for ICU admission, n (%)	18(35)	11(22)	0.157
Length of ICU stay, day (min-max)	3 (1-36)	3.5 (1-18)	0.470
Length of hospital stay, day (min-max)	28 (3-126)	30 (3-130)	0.542
28-day mortality, n (%)	23 (45)	18 (37)	0.395

VAS: Visual analog scale, * Need for intubation and need for noninvasive MV parameters were performed using chi-square test. Other parameters were performed using Mann–Whitney U test.

### 3.2. Secondary outcomes

There was no significant difference in length of ICU and hospital stay between the groups. Twenty-eight-day mortality rate was 45% (23 deaths) and 37% (18 deaths) in HFNC and control groups respectively (p = 0.395) (Table 2). 

## 4. Discussion

Our RCT showed that HFNC compared to oxygen therapy delivered via a mask or nasal cannula applied at hematology and bone marrow transplant wards does not prevent progression of mild acute respiratory failure to endotracheal intubation and mechanical ventilation in hematological malignancy patients. 

Acute respiratory failure is an independent factor on mortality in hematological malignancy patients which indicates the need for developing better management strategies [22]. Mechanical ventilation predicts mortality which may be three times higher in hematological malignancy patients [23]. Preventive measures that can decrease need for mechanical ventilation may decrease mortality in hematological malignancy patients. Early use of noninvasive ventilation during episodes of pneumonitis and hypoxemic acute respiratory failure decreased the need for endotracheal intubation and improved the outcomes in immunocompromised patients [9].

The patients who received noninvasive ventilation had significantly lower rates of endotracheal intubation, complications, mortality compared with patients who received standard treatment with supplemental oxygen [9]. However, there are conflicting results from other randomized studies which showed no benefit from noninvasive ventilation or even increased intubation rates [8,10]. Our study also did not find a significantly reduced intubation rate in hematological malignancy patients. 

HFNC can improve oxygenation in hypoxic respiratory failure and can even decrease mortality rate [16]. A recent meta-analysis comparing efficacy of HFNC and conventional oxygen treatment did not demonstrate a survival benefit compared with conventional oxygen therapy, HFNC use in patients with acute hypoxemic respiratory failure may decrease the need for tracheal intubation [24]. HFNC had no impact on comfort, dyspnea, or ICU/hospital length of stay according to this meta-analysis [24]. However, our study did not find any benefit for intubation rate and comfort of HFNC in immunocompromised patients with respiratory failure although our patients had mild respiratory failure compared to the other studies and our patients were at wards not in intensive care units [16,20,25]. The intubation rate found to be lower in patients with severe hypoxemia (PaO_2_/FiO_2_ < 200) in a post hoc analysis and our group has median PaO_2_/FiO_2_ ratio above 250 which may explain the ineffectiveness of HFNC in our study [16]. Most studies on HFNC in patients with hypoxemia have been conducted in an ICU or high-dependency unit. Authors in a narrative about the mechanism of action and clinical implications of HFNC recommend that HFNC use be limited to ICUs or intermediate care units and its use on regular wards should be discouraged [21]. However, HFNC has been studied in emergency services, postoperative patients and in palliative care units [26–28]. There is limited information HFNC applied at wards in pediatric and adult population [29–31]. HFNC outside the ICU was associated with improved visual analog scale score, breathing frequency, and saturation but with a relatively high mortality, care should be exercised in using this therapy in a setting that is not continuously monitored [31]. Our hematology and bone marrow transplantation units have continuous pulse oximetry and ECG monitoring on demand of the caring team. The patients were transferred to the ICU as they required per caring team. Patients required HFNC at wards should have low threshold for ICU transfer or HFNC treatment should be applied in the ICU. 

Our 28-day mortality rate was similar with other studies [20] although our patients have less severe respiratory failure. It was shown that delay in ICU management is associated with mortality [32]. Our patients were followed at hematology ward which may cause delay to ICU admission although we had certain criteria for treatment failure in our patients. Invasive and noninvasive mechanical ventilation more often required in hematological malignancy patients with respiratory failure [33]. We observed significant number of our patients progressed from mild respiratory failure to more severe form and required invasive and noninvasive mechanical ventilation.

Dryness and discomfort were found to be significantly lower in the HFNC patients compared standard nonhumidified oxygen therapy in a randomized trial in patients with ARF who did not require immediate NIV or MIV [13]. However, these patients were not immunocompromised and less severe patients. Comfort, thirst and dyspnea VAS scores were not different with HFNC and standard oxygen treatment groups in a group of immunocompromised hypoxemic respiratory failure patients as similarly with our study [34]. 

Our study has several limitations. Firstly, limited number of the patients and we did not perform power analysis prior to the study. Secondly, therapies were not blinded. Thirdly, since we performed the study in a ward, the results are not applicable to the patients in intensive care units. Finally, limited statistical power because of the modest sample size in the present study (n = 100) may have played a role in limiting the significance of some of the statistical comparisons conducted. A post hoc power analysis revealed that on the basis of the mean, between-groups comparison effect size observed in the present study (d = 0.29), an n of approximately 358 would be needed to obtain statistical power at the recommended 0.80 level [35].

## 5. Conclusion

In hematological malignancy patients with mild acute respiratory failure HFNC compared to oxygen treatment delivered via a mask or nasal cannula did not improve the need for ICU transfer, requirement for either noninvasive mechanical ventilation or invasive mechanical ventilation. There were also no significant differences in VAS scores for dyspnea, comfort, and thirst between both treatment groups. The requirement of invasive and noninvasive mechanical ventilation, length of hospital stay, ICU stay, and 28-day mortality between HFNC and oxygen delivered via a mask were similar. Standard medical treatment and oxygen support with mask or nasal cannula must be main approach to mild respiratory failure patients with hematological malignancy who are treated at wards. 

## Author contributions

N.A.M., S.T., R.C.Y. and M.S. had access to all of the data in the study and take responsibility for the integrity of the data and content of the manuscript N.A.M., S.T., R.C.Y., B.E., L.G.K., K.G, and M.S. contributed substantially to the study design, data analysis and interpretation, and writing and review of the manuscript.
